# Lupus Vulgaris Treated by Direct Sunshine

**Published:** 1908-12-05

**Authors:** 


### LUPUS VULGARIS TREATED BY DIRECT SUNSHINE.

There is a very general tendency, especially in the female sex, to avoid direct exposure of the face to the sun's rays if the latter are at all strong; and yet there can be no doubt of the health-giving properties of direct sunshine. How sunshine acts in this respect it is difficult to say; it is possible that, in addition to any local chemical changes produced at the site of impact of the rays, there are substances elaborated and carried away to other parts of the body, influencing metabolism generally and enabling the tissues to cope with and throw off bacterial and other diseases. Fresh air is much, but fresh air and sunshine together are far more. There is perhaps a tendency to lay too little stress upon the sunshine.

The effects of the sun's rays in the cure of local diseases of the skin, especially lupus vulgaris, is better recognised nowadays than it used to be; but even here there is a general tendency to regard a special apparatus and the Finsen light as essential. There are many cases in which Finsen light treatment cannot be procured either on account of the locality in which the patient lives, the absence of any apparatus in any neighbouring town, or on account of the expense. A good deal can be done for such cases even at home by making the very most of direct exposures to the unscreened rays of the sun, whilst for patients who can afford to go to places where the sun shines more hotly and more constantly than it does in Great Britain, very striking results can be obtained. Dr. J. Goodwin Tomkinson, of Glasgow, drew attention to this in a paper before the 1908 meeting of the British Medical Association; and in France the treatment of lupus vulgaris by direct sunshine has been successfully carried on for some years. M. Revillet and M. Widal independently communicated a whole series of cases to the Paris Congress on Tuberculosis in 1905.

The essential part of the technique is that the patients shall be exposed so that the sun's rays fall as directly as possible upon the lesions for at least two or three hours a day, beginning perhaps with less than this, according to the sensitiveness of the healthy cutis, but ultimately increasing to seven or eight hours a day, when the sun-burning and consequent pigmentary protection have become considerable. Sunstroke is avoided by the wearing of a suitable light hat, and the eyes are protected by smoked or blue glass spectacles; but the brim of the hat must be arranged so that it does not shade the part treated nor its immediate neighbourhood.

The cases reported to the Paris Congress on Tuberculosis in 1905 consisted both of lupus vulgaris and of scrofulodermia as distinct from the former. Naturally the scrofulodermia patients were relieved more readily than were those suffering from lupus; but nearly three-quarters even of the latter were cured, at any rate for the time being. Dr. Tomkinson records a case which very strikingly shows how much can be effected by systematic exposure to unmitigated sun rays even in a very chronic case that has resisted other forms of treatment. The patient was a lady who had had lupus vulgaris for 23 years. It had begun quite early in childhood, and now affected both cheeks extensively, reaching also into both submaxillary regions. Much of the affected area was cicatrised, but typical apple-jelly nodules were present, disseminated over both sides of the face, and there was no doubt of the activity of the disease process, notwithstanding every form of treatment, including Finsen light and tuberculin injections. The patient being in the habit of wintering in Egypt, it was suggested that she should take the opportunity to adopt heliotherapy. She did so persistently for four months, and only desisted when she left Egypt to return home. During these four months the face had been exposed to the full blaze of the Egyptian sun (January to May) for an average of five hours a day, with the head and eyes protected in the way described above. At first there was a reactionary dermatitis, six blisters developing upon the right cheek. It is probable that, with more graduated exposures, any serious or painful dermatitis could be avoided without the cure of the lupus being made any less sure; a simple lotion of zinc oxide, boric acid, and calamine soon relieved the dermatitis.

On returning to Scotland after the four months' heliotherapy it was found that no apple-jelly nodules were to be detected; the scar tissue was supple and good; the most that could be discerned in the way of activity in the lupus were one or two faintly hyperæmic macules. The patient's general health had greatly improved at the same time.

